# Larva and pupa of Ctesias (s. str.) serra (Fabricius, 1792) with remarks on biology and economic importance, and larval comparison of co-occurring genera (Coleoptera, Dermestidae)

**DOI:** 10.3897/zookeys.758.24477

**Published:** 2018-05-15

**Authors:** Marcin Kadej

**Affiliations:** 1 Department of Invertebrate Biology, Evolution and Conservation, Institute of Environmental Biology, Faculty of Biological Science, University of Wrocław, Przybyszewskiego 65, PL–51–148 Wrocław, Poland

**Keywords:** exuvia, immature stage, larva, pupa, seta, terga

## Abstract

Updated descriptions of the last larval instar (based on the larvae and exuviae) and first detailed description of the pupa of Ctesias (s. str.) serra (Fabricius, 1792) (Coleoptera: Dermestidae) are presented. Several morphological characters of *C. serra* larvae are documented: antenna, epipharynx, mandible, maxilla, ligula, labial palpi, spicisetae, hastisetae, terga, frons, foreleg, and condition of the antecostal suture. The paper is fully illustrated and includes some important additions to extend notes for this species available in the references. Summarised data about biology, economic importance, and distribution of *C. serra* are also provided. The comparison of larval characteristics for some of the genera of Dermestidae co-occurring with *Ctesias* is presented. A key for identification of these genera is also provided.

## Introduction

The genus *Ctesias* Stephens, 1830 is placed in the tribe Megatomini in the subfamily Megatominae. According to the world catalogue of Dermestidae, the genus contains only 26 species (Háva 2018) distributed mainly in the Palaearctic and Afrotropical zone (Háva 2015, 2018). They all have been divided into four subgenera, *Ctesias* Stephens (four species), *Decemctesias* Háva (fourteen species), *Novemctesias* Háva (two species) and *Tiresiomorpha* (Pic) (six species) (Háva 2018). The genus seems to be closely related to genera such as *Globicornis* Latreille, 1829, *Megatoma* Herbst, 1791, *Trogoderma* Dejean, 1821 and *Reesa* (Milliron, 1939), but can be distinguished in the adult phase by the following characteristics: sharply defined antennal cavity and well developed antennal club with subtriangular antennomeres, giving the male antennal club a serrate appearance (Mroczkowski 1975, Peacock 1993). The larval features that distinguish *Ctesias* from related genera like *Globicornis*, *Megatoma*, *Trogoderma* and *Reesa* were given by Peacock (1993) and are mainly expressed by location of tufts of the hastisetae (= dense brushes of hastisetae) on the abdominal terga. In *Ctesias* these are located on each side of a membrane behind the tergum and moreover abdominal segment VIII lacks tufts. In comparison in *Globicornis*, *Megatoma*, *Trogoderma* and *Reesa*, tufts of hastisetae are situated on sclerotised areas of terga and never on membranes behind the terga (i.e., hastisetae are concentrated on the lateral portions of the posterior abdominal terga, behind the row of stout spicisetae); abdominal segment VIII with tufts of hastisetae. Due to the presence of the tufts of hastisetae on membranous emarginations of the terga, larvae of *Ctesias* are similar to *Anthrenus* Geoffroy, 1762 with the main difference between these genera being the shape of the body. In *Ctesias* the body is constricted behind the abdominal terga I–III, which are each longer than tergum IV, while in *Anthrenus* the body is not constricted and usually widened evenly from the pronotum (broadest at abdominal terga IV–VI (Beal 1991)), according to Peacock (1993) II–V. Moreover, in *Ctesias* there are four tufts of hastisetae (on membranous areas behind the abdominal terga IV–VII), while in *Anthrenus* there are only three (on the membranous areas behind the abdominal terga V–VII).

Interestingly, of the 21 species of *Ctesias*, the larval stages of only one, *Ctesias serra* (Fabricius, 1792), are referred to in the literature (compare with Table 1). In this paper, an updated description of larva of *Ctesias serra* (Fabricius, 1792) is given. This species represents the nominal subgenus Ctesias s. str. and is widely distributed in Europe. It has been also recorded from Algeria, Russia, and Caucasus (Háva 2015). The species is widely distributed through most European countries and is associated with areas of mature trees such as old parks, ancient woodlands, pasture woodlands, hedgerow trees, and forests (Peacock 1993). The paper presents some additions to extend notes for this species available in the references. The following set of larval characters are described and illustrated for the first time: foreleg, frons, pronotum, abdominal segment I, abdominal segments VII–IX. Additonally, the pupa is described and illustrated for the first time. Summarised data about the biology, economic importance, and distribution of *C. serra* are also provided.

The current work is a continuation of the previous articles devoted to study the morphology of the immature stages of Dermestidae (Beal and Kadej 2008, Kadej 2012a, b, c, Kadej and Jaroszewicz 2013, Kadej et al. 2013a, b, Kadej and Guziak 2017a, b, Kadej 2017, Kadej et al. 2017).

**Table 1.** List of *Ctesias* species with references related to larval morphological characters.

Taxa	References	Available data
*Ctesias* Stephens, 1830	Lepesme and Paulian 1939	Short sentence in key (p. 167)
	Beal 1967	Short sentence in key (p. 290)
	Klausnitzer 1978	Short sentences in key (p. 168) [in German]
	Peacock 1993	Short sentences in key (p. 37)
	Klausnitzer 2001	Short sentences in key (p. 31–32) [in German]
Ctesias (s. str.) serra (Fabricius, 1792)	Perris 1846	Brief description of larval morphology (p. 339) [in French], illustration of larval habitus, antenna, setae (p. 345, pl. IX, fig. 4f)
	Decaux 1891	Short description of larval morphology (p. 27) [in French] and illustration of habitus (p. 27)
	Donisthorpe 1897	Brief description of larval morphology (p. 162), pupa (p. 162)
	Böving and Craighead 1931	Illustration of apex of maxilla (p. 267), mouthparts (ventral, p. 267), habitus (lateral view, p. 267)
	Lepesme and Paulian 1939	Illustration of antenna (p. 163)
	Rees 1943	Short sentence in key (p. 7), brief description of larval morphology (p. 12), and illustration of antenna (p. 15), epipharynx (p. 18)
	Korschefsky 1944	Short description in key (p. 150), illustration of habitus (p. 154, pl. II)
	Klausnitzer 1978	Short sentence in key (p. 168) [in German], illustration of habitus (p. 169)
	Peacock 1993	Short description in key (p. 37) and on pages 43, 60, illustration of larval habitus (p.120), epipharynx (p. 128) [epipharynx shown after Rees (1943)]
	Klausnitzer 2001	Illustration of habitus (dorsal, lateral view, p. 34), antenna (p. 34), epipharynx (p. 34) [habitus and epipharynx shown after Peacock (1993)]

## Materials and methods

For morphological examination of larvae and exuviae of the last-stage, specimens stored in ethanol were used. The material came from the collection of the Department of Invertebrate Biology, Evolution and Conservation, University of Wrocław (DIBEC). Larva/exuvium were boiled for 3–10 minutes in 10% solution of KOH, and then rinsed with distilled water. They were then placed in distilled water for ~1 hour to clean and soften the material. All structures were put in glycerin on slides. The morphological structures were studied under a Nikon Eclipse E 600 phase contrast microscope with a drawing tube attached, and a Nikon SMZ-800 binocular microscope; examination was done using transmitted light. Photos were taken with Canon 500D and Nikon Coolpix 4500 cameras under Nikon Eclipse 80i and/or Nikon SMZ-800. In addition to the description provided herein, plates of the larval habitus/pupa as well as drawings of selected elements are also provided.

The terminology used in this paper follows Kiselyova and McHugh (2006), Kadej and Jaroszewicz (2013), and Kadej et al. (2013a, b).

### Figure abbreviations

**ac** acrotergite;

**as** antecostal suture (ridge);

**asg** abdominal segments;

**b** transverse row of placoid sensillae on epipharynx;

**c** claw;

**cs** campaniform sensilla;

**dst** distal epipharyngeal sensillae;

**dmr** dorsomesal row of setae on lacinia;

**er** epipharyngeal rods;

**f** femur;

**fe** fore wing;

**g** galea;

**hw** hind wing;

**l** lacinia;

**lp** labial palp(i);

**mp** mesal pair of labor-epipharyngeal setae;

**ms** mesonotum;

**msr** mesal row of setae on lacinia;

**mt** metanotum;

**mxp** maxillary palp(i);

**p2** second pair of labor-epipharyngeal setae;

**pls** placoid sensilla;

**pr** pretarsus;

**pro** pronotum;

**prs** processes;

**s** sensorium (accessory sensory papillae);

**sbp** subproximal epipharyngeal sensillae;

**st** stipes;

**t** tibia;

**tr** tubercula.

## Taxonomy

### Subfamily Megatominae Leach, 1815

#### Tribe Megatomini Ganglbauer, 1904

##### Genus *Ctesias* Stephens, 1830

###### Ctesias (s. str.) serra

Taxon classificationAnimaliaColeopteraDermestidae

(Fabricius, 1792)

Figs 1–4 , 5–6 , 7–15 , 16–21

####### Material examined.

(2 larvae) Polonia, Brzóza distr. Kozienice 7.VII.1956, w próchnie pnia lipy [inside the mould of the trunk of linden *Tilia* spp.], leg. B. Burakowski, det. M. Mroczkowski 1956; (2 larvae) Polonia, Maciejowice distr. Kozienice 6.VII.1956, w próchnie (bielu) dębu koło chodników Anobiidae [inside the mould of the oak *Quercus* spp. next to corridors of Anobiidae], leg. B. Burakowski, det. M. Mroczkowski 1956; (1 larva) Polonia, Maciejowice distr. Kozienice 6.VII.1956, pod korą olchy [under the bark of alder *Alnus* spp.], leg. B. Burakowski, det. M. Mroczkowski 1956; (7 larvae) Puszcza Kampinoska, Sieraków, 31.X.1952, pod kora dębu [under the bark of the oak *Quercus* spp., leg. M. Mroczkowski]; (1 exuvia, 1 pupa) Warszawa, Saska Kępa pod korą wierzby [under the bark of willow *Salix* spp.] 10.V.1955, leg. M. Mroczkowski; (1 exuvia, 4 larvae) Polonia, Dojlidy ad. Białystok 19.III.1959 leg. R. Bielawski, det. M. Mroczkowski 1959; (1 larva) Germania: Brandenburg, Berlin, Schorfheide, 1.IV.1994. leg. A. Herrmann, coll. A. Herrmann. All materials (except for the last larva) are deposited in the Department of Invertebrate Biology, Evolution and Conservation, University of Wrocław, Przybyszewskiego 65, PL–51–148 Wrocław, Poland.

####### Description.

**Figures 1–4.**
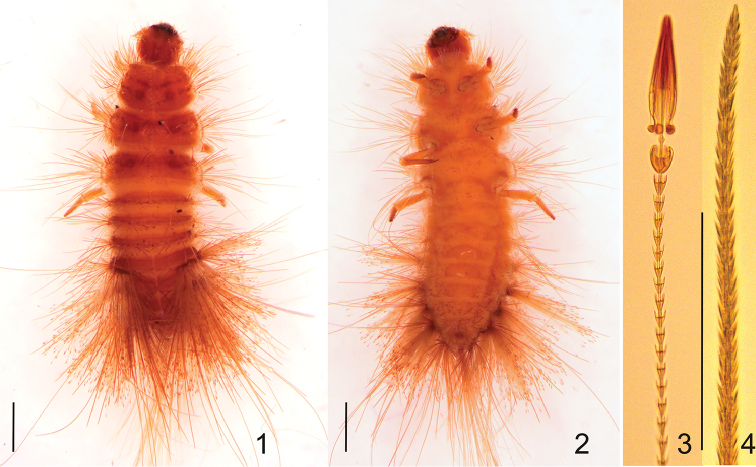
Mature larva of Ctesias (s. str.) serra (Fabricius, 1792). **1** Dorsal view **2** Ventral view **3** Head (apex) of hastiseta **4** Spiciseta. Scale bars: 0.1 mm.

Larva, last instar. Length 5.0–7.0 mm. Body fusiform, relatively long, rather flattened, not hunchbacked. Integument of head, nota and terga yellowish brown to brown; tergal plates sclerotised (Fig. 1), sterna only partially hyaline (= sterna I–VIII with central median line with strongly sclerotised and shiny strip (Fig. 2)), femora and tibiae light yellowish (Figs 1–2). On thoracic terga (= nota I–III) there are darker spots or patches present. Setae (spicisetae and hastisetae) on tegra brown (Fig. 1). On sterna only brown scaly-like spicesetae present (Fig. 2). Head protracted and hypognathous. Six stemmata present on the head (four + two other below). Frons triangular, without frontal, median tubercle (Fig. 8). On the frons two kinds of spicisetae present: lanceolate (= nudiseta) and scale-like. Lanceolate setae situated along anterior margin and on the central area, while scaly spicisetae along lateral margins and in the posterior part of the plate; several also present on the central area among lanceolate setae. Antennae orientated anterolaterally; composed of three antennomeres (Fig. 7). Terminal antennomere 4.0 times as long as wide, with one small sensory sensillum (appendage) on apex and two campaniform sensillae (upper one small, lower one bigger). Ratio of length of terminal antennomere to length of penultimate and antepenultimate antennomeres combined nearly 1.0:5.0. Sensorium in ventral position not extending above apex of segment 2. One campaniform sensillum present on antennomere 2 under sensorium. Antennomere 1 with 6–7 long setae (Fig. 7). Gula separate from postmentum; epicranial stem present. Median endocarina absent. Labro-epipharyngeal margin with 8 to 11 setae in the outer series. Mesal labro-epipharyngeal setae (mp) spatulate (broad) while second pair (p2) stout (narrow). On ventral side of epipharynx basal transverse row (br) of placoid sensillae present (13 to 18 sensory cups in the proximal transverse series (br)). Epipharyngeal rods (er) present and diverging proximally. Four sensory cups in the subproximal epipharyngeal sensilla (sbp), two large and two small ones. Distal epipharyngeal sensillae (dst) arranged in one group of six (in two longitudinal series of three sensillae, Fig. 11). Lateral setae on epipharynx absent (Fig. 11). Dorsal surface of labro-epipharynx with many setae. Mandible brown with dark brown (almost black) apex; apical teeth and ventral accessory process absent. Apical half of mandible heavily sclerotised and sharply delineated from basal half (Figs 9, 10). Mandibular mola and pseudomola absent. Hyaline lobe at ventral base of mandible absent. Prostheca perhaps absent, brush of setae absent mesally near mandibular base. Placoid sensillae (pls) present in approximately one-third of the basal dorso-lateral length of mandible (Fig. 10). Maxillary palp composed of three palpomeres with terminal palpomere longest. Ratio length of terminal palpomere to length of the two proceeding palpomeres combined 1.0:1.5. First palpomere with variable combination of setae and campaniform sensilla:, two setae (one campaniform sensillum) or four setae (one campaniform sensillum). Second palpomere with 2–3 setae and 1–4 campaniform sensillae. Third palpomere with one campaniform sensillum, one short seta subapical and group of small sensillae situated in the apical area. Lacinia with one heavily sclerotised lacinial tooth, straight at apex. Lacinia sclerotisation separated from stipes. Seven straight thick to slender setae present in a dorsomesal row on lacinia (dmr) (Fig. 12). Mesal row of setae on lacinia (msr) composed of a basally thickened seta (Fig. 13). Galea arising from stipes, ending close to the apex of lacinia. The apical area of galea covered densely with setae. Stipes with 18–20 long setae placed mainly near the antero-lateral margin, one to two setae present near the inner margin (under the first palpomere) (Fig. 13). Hypopharynx hyaline. Bridge sclerite (central part of the distal element of the hypopharyngeal sclerome) appearing jointed medially. Anterior arms of bridge sclerite and distal lateral sclerites of hypopharynx absent. Ligula with approximately 21 lanceolate setae (Fig. 14). Labial palp with 2 palpomeres. First segment wider than second segment; 2.0 times as wide as long, with four setae on the disc (sometimes setae absent – they can be lost during dissection - then resembling campaniform sensillae). Terminal labial palpomere with group of small sensillae in the apical area, one campaniform sensillum (cs) close to external margin and three setae on inner margin (Fig. 15).

**Figures 5–6.**
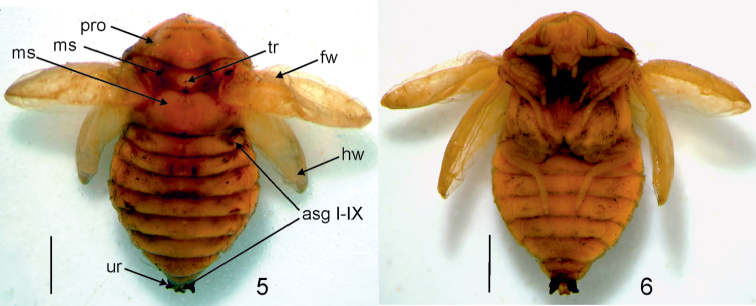
Pupa of Ctesias (s. str.) serra (Fabricius, 1792). **5** Dorsal view **6** Ventral view Scale bars: 0.1 mm.

**Figures 7–15.**
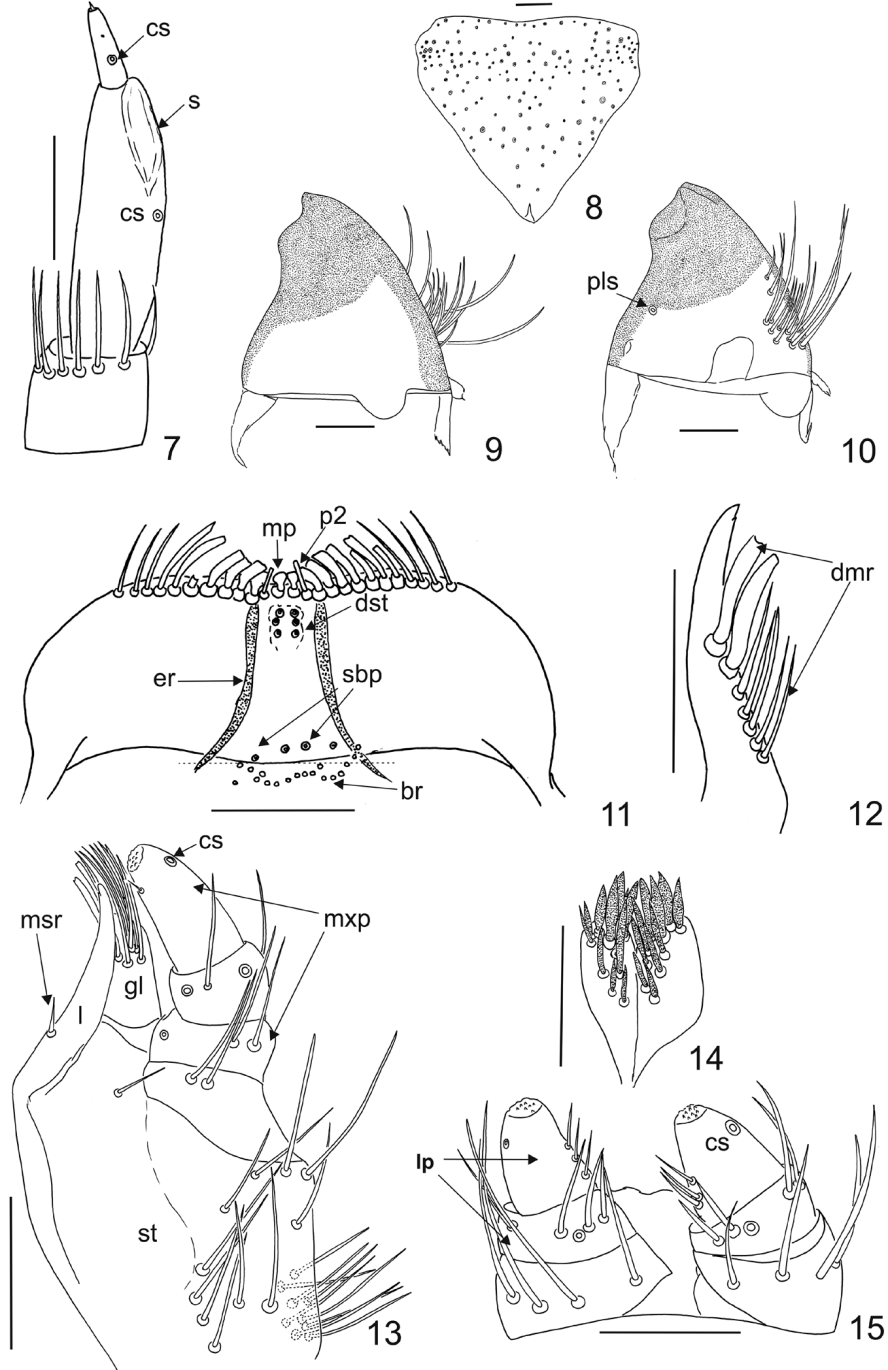
Mature larva of Ctesias (s. str.) serra (Fabricius, 1792). **7** Antenna (dorso-fronto-lateral) **8** Frons (dorsal; large circles with rings represent points of insertion of large scaly-like spicisetae, small circles represent points of insertion of nudisetae (= lanceolate spicisetae)) **9** Mandible (dorsal) **10** Mandible (lateroventral) **11** Epipharynx (ventral) **12** Apex of lacinia (dorsal) **13** Maxilla (ventral) **14** Labium (ventral) **15** Labial palpi (ventral). Scale bars: 0.1 mm.

**Figures 16–21.**
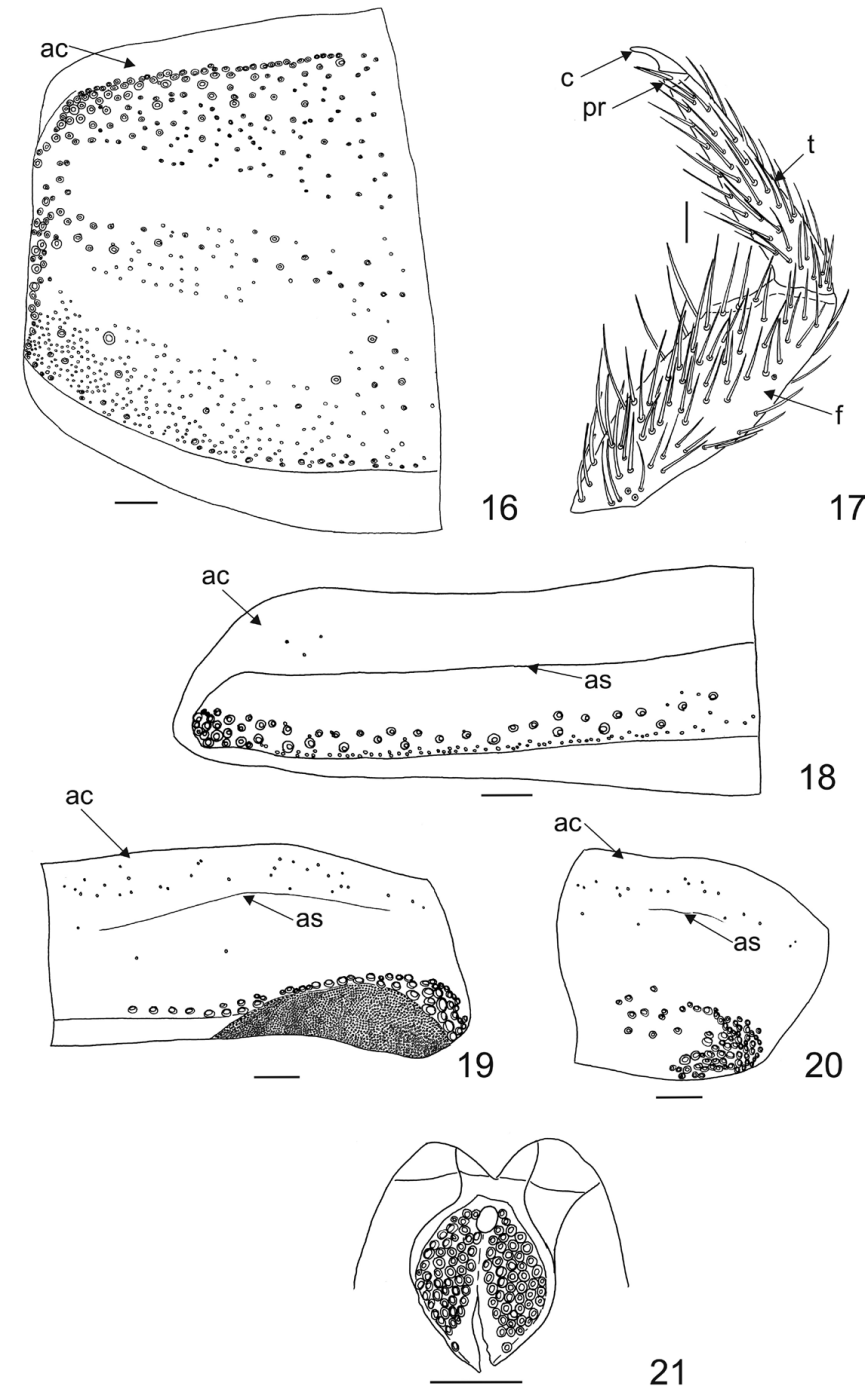
Mature larva of Ctesias (s. str.) serra (Fabricius, 1792). **16** Pronotum (dorsal, left half, denuded; large circles represent points of insertion of large spicisetae, small circles along the suture represent points of insertion of hastisetae) **17** Right protosternal leg (dorsal) **18** Abdominal tergum I (dorsal, left half, denuded; large circles represent points of insertion of large spicisetae, small circles represent points of insertion of hastisetae) **19** Abdominal tergum VII (dorsal, right half, denuded; large circles represent points of insertion of large spicisetae, small circles along the suture represent points of insertion of short setae, small circles below large circles represents points of insertions of hastisetae) **20** Abdominal tergum VIII (dorsal, right half, denuded; large circles represent points of insertion of large spicisetae, small circles along the suture represent points of insertion of short setae) **21** Abdominal tergum IX (dorsal, denuded; circles represent points of insertion of large spicisetae). Scale bars: 0.1 mm.

Antecostal suture on notum I absent, but distinct on nota II–III and abdominal terga I–VII (Figs 18 and 19); abdominal segment VIII without suture or only remnant remaining (Fig. 20). Acrotergites of notum I without setae, while acrotergites of nota II–III and abdominal terga I–VIII with short setae (Figs 18–20). Notum I with long, stout, large spicisetae along anterior (here directed anteriorly under the head), lateral and posterior margin (here directed latero-posteriorly and vertically - upright). Setae on posterior margin situated near the latero-posterior angle, some additionally near suture, some also present on central area of disc of notum I (Fig. 16). Nota II, III with median row of large spicisetae, and along lateral margins of terga. Abdominal terga I–VII with posterior rather than median row of large spicisetae, and along lateral margins of terga (Figs 18–19). These mainly directed latero-posteriorly and vertically (upright). Hastisetae are present both on nota as well as abdominal terga (Figs 16, 18–20). Hastisetae of abdominal terga IV–VII forming dense lateral brushes (longest and thickest on V–VII). Setal patterns of abdominal tergum I with numerous large spicisetae in posterior row; lateral margin bearing also spicisetae; hastisetae on posterior half of tergite more numerous than spicisetae (Fig. 18). Abdominal tergum VII with short, stout setae along anterior margin; large spicisetae in posterior row above the membranous area bearing densely situated hastisetae (Fig. 19). Abdominal tergum VIII without pair of abdominal pits (oval apertures); setal patterns as illustrated (Fig. 20) - short, stout setae along anterior margin; large spicisetae in posterior part. Abdominal tergum IX reduced with numerous long scaly-like spicisetae (Fig. 21). Legs (tibia, femur and trochanter) covered with many lanceolate setae as illustrated on Fig. 17. Claws dark brown. Ratio tibial to femoral length 4.0:5.0. Pretarsus with two narrow lanceolate setae inserted at base. Length of posterior pretarsal seta subequal to length of anterior pretarsal seta (Fig. 17), anterior pretarsal seta perhaps slightly longer.

Pupa (Figs 5–6): length 4.0–5.0 mm. Integument yellowish brown with erect, brown coloured spicisetae distributed rather uniformly on head, dorsum and wings. Head directed downwards (not visible from above). Antennae long, reaching lateral margin of pronotum. Antenna with 11 antennomeres (the boundaries of individual segments not sharply delimited); antennal club with 3 antennomeres (Fig. 6). Antennal club serrated, shorter than flagellum. Eyes clearly visible, convex, oval; situated just behind upper margins of antennae. Pronotum transverse, widest near mesonotum (between posterior angles), with the anterior part narrowed; posterior border of pronotum distinctly elongated in the middle; posterior angles slightly rounded. Mesonotum half as long as metanotum. Mesonotum and metanotum slightly convex. Mesonotum with distinct tubercula in the central part of the disc. Hind wings shorter than fore wings, reaching posterior margin of abdominal segment IV (Fig. 5). The width of abdominal segments I–IV gradually broadened, while V-VIII narrowed posteriorly (Fig. 5). Abdominal segment IX with two black processes (Figs 5–6) (from lateral view these processes slightly curved upward). Abdominal segment IX emarginated in the middle. Legs visible, well developed. Gin traps absent (Fig. 5). Pupa remains within the last exuvium (= larval skin) which is interrupted from head to last abdominal terga (Donisthorpe 1897, 1920). Probably pupa anchored by two clusters of long fine setae inserted on each side of the abdominal tergum VIII.

####### Biology.

Knowledge of the biology of the species is limited, with only a small amount of published information (Donisthorpe 1920, Mroczkowski 1975, Peacock 1993). There is probably only one generation a year. In Poland, adults occur from May to July and sometimes August. Beetles have been recorded from under bark, from tree cavities, by sap flows, and on flowers (where they usually copulate). The eggs are laid under the bark of trees and usually number ca. 20-40. The larvae hatch after 2-3 weeks, passing through usually five instars. Pupation takes place in Autumn or Spring (in April). Since both larvae of the last instar and pupae have been observed under bark throughout the winter, it appears that the species can overwinter as either a pupa or larva. It is known that larvae live under the bark of the mature trees (of different species such as: oak, poplar, elm, sycamore, hawthorn, fir, beech, horse and sweet chestnut, maple, redwood, cherry and willow), close to spiders’ webs, where they feed on dead insects (Rees 1946, Burakowski et al. 1986, Peacock 1993, Kadej 2005). They also feed on clutches of butterflies eggs (Mroczkowski 1975, Peacock 1993). Occasionally, larvae have been observed in the nests of Aculeata, where they feed on the larval exuviae. They have also been found in insect galleries (e.g. cerambycid *Nothorhina punctata* (Fabricius, 1798)), in old fungus, and in rotting trees and stumps of mainly deciduous trees (Hämäläinen and Mannerkoski 1984, Peacock 1993). Due to a secretive life they are usually observed as immature stages. The larvae, when disturbed by predators, can erect and vibrate the abdominal brushes of hastisetae (Donisthorpe 1897, Joy 1920, Rees 1946). This specific way of defence is facilitated by a well-developed supra-anal organ on the last abdominal segment (Mroczkowski 1975, Peacock 1993).

####### Economic importance.

Probably because of its rarity, this species has no serious economic importance. However, it is likely that in its natural habitat it can play a positive role in reducing the number of eggs of butterflies classified as pest of forests (Mroczkowski 1975). Harding (1986) classified the species as an old forest indicator.

####### Distribution.

Widely distributed in Europe (from the Mediterranean region to the UK and the southern province of Fennoscandia). It has been also recorded from Algeria, Caucasus and Russia (Stavropol) (Háva 2015).

## Discussion

Most of the larval morphological characteristics presented here are shown for the first time for *C. serra*. In comparison with previous papers of Perris (1846), Böving and Craighead (1931), and Lepesme and Paulian (1939), the current graphics illustrate much more detailed larval morphology. Some of the graphics used by previous authors are reproduced images (e.g. Rees (1943) or Peacock (1993)). Thus based on the available data, only superficial comparisons were possible. Moreover, some characteristics were mistakenly interpreted by previous workers. For instance, Perris (1846, p. 310) wrote that the antenna of *C. serra* has 4 segments, while actually there are only 3.

However, the morphology of epipharynx is quite interesting. Structures shown by Rees (1943, p. 18), Peacock (1993, p. 128) and Klausnitzer (2001, p. 34) are similar to those studied by me (compare with Fig. 11, current paper) with the exception of complexes of the sensillae (sbp) and (br). I have observed more placoid sensillae in the basal transverse row (br). There were 13 to 18 sensory cups in the proximal transverse series (br), whilst in abovementioned papers there were only 10. I also recorded four sensory cups in the subproximal epipharyngeal sensilla (sbp) – two big and two small ones, while Rees (1943, p. 18), Peacock (1993, p. 128) and Klausnitzer (2001, p. 34) observed only two.

It is difficult to compare larval characters of *C. serra* with congenerics because of a lack of larval morphological descriptions for the other species. For this reason I decided to collect and summarise larval data for the genera which co-occur with *Ctesias* (see Table 2). The aim was to focus on these larval characteristics which allow for easy identification at the generic level.

**Table 2:** Comparison of larval characteristics for some of the genera of Dermestidae co-occurring with *Ctesias serra*.

Character	Body shape	Colour of integument	Tegites/Sternites	Urogomphi	Abdominal tufts of hastisetae
Genus					
***Dermestes* Linnaeus, 1758**	Elongate, cylindrical (gradually tapering to last abdominal segment; ratio length to width 1.0:4.5); broadest at notum III; without caudal brush of long, slender setae	Usually dark brown to black, but sometimes yellowish and sometimes with median yellowish strip from anterior margin of pronotum	Tergites usually strongly sclerotised. Sternites membranous (= hyaline), but those of abdominal segments IX-X, and occasionally VII-X, entirely sclerotised	Present on abdominal tergite IX dorsally	Absent
***Thylodrias* Motschulsky, 1839**	Compact and C-shaped, cyphosomatic (ratio length to width 1.0:3.0); broadest at abdominal segment I; without caudal brush of long, slender setae	Light golden brown	Tergites sclerotised. Sternites membranous (= hyaline)	Absent	Absent
***Trinodes* Dejean, 1821**	Relatively short (= compact, ratio length to width 1.0:2.5); broadest at abdominal segment I; without caudal brush of long, slender setae	Greyish with sclerotised brown strip along anterior and posterior margin of tergite, enclosing a transverse membranous area on each side	Tergites sclerotised. Sternites membranous (= hyaline)	Absent	Absent
***Attagenus* Latreille, 1802**	Elongate, cylindrical, orthosomatic (gradually tapering to last abdominal segment; ratio length to width 1.0:6.0); broadest at notum I; with caudal brush of long, slender setae	Yellowish brown to brown	Tergites sclerotised. Sternites membranous (= hyaline)	Absent	Absent
***Anthrenus* Geoffroy, 1762**	Relatively short (= compact, ratio length to width 1.0:2.5); broadest at abdominal terga IV–VI^1^; flattened, not hunchbacked and not constricted; slender setae present on tergum IX, but not so long as in *Attagenus*, *Ctesias, Megatoma*, *Reesa*, *Trogoderma*	Yellowish brown to dark brown; sometimes with darker spots or patches on terga	Tergites sclerotised. Sternites sometimes sclerotised	Absent	On membranous area behind terga V–VII
***Ctesias* Stephens, 1830**	Fusiform, and relatively long, sub-oblong (ratio length to width 1.0:3.0); broadest at notum III; rather flattened, not hunchbacked; constricted behind abdominal terga I–III; with caudal brush of long, slender setae	Yellowish brown to brown; thoracic terga (= nota I–III) sometimes with darker spots or patches	Tergites sclerotised. Sterna I–VIII with central median line with strongly sclerotised and shiny strip	Absent	On membranous area behind terga IV–VII
***Globicornis* Latreille, 1829**	Fusiform, and relatively long, sub-oblong (ratio length to width approx. 1.0:4.5); broadest at notum III; slightly hunchbacked; with caudal brush of long, slender setae	Brown to dark brown	Tergites sclerotised. Sternites membranous (= hyaline)	Absent	On terga V–VIII^2^ (never on membranes behind terga)
***Megatoma* Herbst, 1792**	Fusiform, and relatively long (ratio length to width 1.0:4.5); broadest at notum III; flattened, not hunchbacked; with caudal brush of long, slender setae	Yellowish brown (in some species thoracic terga I–III with distinctly dark brown patches at sides, sometimes extending to middle on terga II and III)	Tergites sclerotised. Sternites membranous (= hyaline)	Absent	On terga VI –VIII (never on membranes behind terga)
***Reesa* Beal, 1967**	Fusiform, and relatively long (ratio length to width 1.0:4.0); broadest at notum III; rather flattened, not hunchbacked, orthosomatic; with caudal brush of long, slender setae	Yellowish brown to dark brown (then strongly pigmented)	Tergites sclerotised. Sternites membranous (= hyaline)	Absent	On terga I–VIII (but the longest and thickest on VI–VIII) (never on membranes behind terga)
***Trogoderma* Dejean, 1821**	Fusiform, and relatively long (ratio length to width 1.0:2.0); broadest at notum III; rather flattened, not hunchbacked, orthosomatic; with caudal brush of long, slender setae	Yellowish brown to dark brown	Tergites sclerotised. Sternites membranous (= hyaline)	Absent	On terga V(VI)–VIII (longest and thickest on VI–VIII) (never on membranes behind terga)

^1^ According to Beal (1991) body of *Anthrenus* larvae is broadest at abdominal segments IV–VI, while according to Peacock (1993) at abdominal segments II–V. ^2^ In *G. corticalis* and *G. emarginata*, but it is likely that this feature is similar also in *G. nigripes*.

**Table 2.2:** Continuation of the comparison of larval characteristics for some of the genera of Dermestidae co-occurring with *Ctesias serra*.

Character	Body setation	Ratio length to width of head of hastiseta	Antecostal suturae	Epipharynx	Setae Mp/p2 on labro-epipharyengal margin
Genus					
***Dermestes* Linnaeus, 1758**	Hastisetae absent (only spicisetae present, occasionally modified into ramous setae or club-shaped setae)	N/A	Distinct, present on nota II–III and abdominal terga I–X	No distal epipharyngeal sensilla (dst)	Middle 4 setae of labro-epipharyengal margin consisting of 2 spatulate, broad inner (Mp) and 2 stout, narrow (p2) outer setae
***Thylodrias* Motschulsky, 1839**	Hastisetae absent (club-shaped setae present^3^; spinulate setae only on transverse membranous areas of each tergite, not on pronotum)	N/A	Distinct, present on nota II–III and abdominal terga I–VIII	No distal epipharyngeal sensilla (dst)	Middle 4 setae of labro-epipharyengal margin consisting of 4 spatulate, broad setae both in inner (Mp) and (p2) outer setae
***Trinodes* Dejean, 1821**	Hastisetae absent (black, erect spicisetae present)	N/A	Distinct, present on nota II–III and abdominal terga I–VIII	No distal epipharyngeal sensilla (dst)	Middle 4 setae of labro-epipharyengal margin consisting of 2 spatulate, broad inner (Mp) and no setae (p2) in outer series^4^
***Attagenus* Latreille, 1802**	Hastisetae absent (only spinulate (= lanceolate) and in some species also scale-like setae present)	N/A	Distinct, present on nota II–III and abdominal terga I–VIII	Usually 2 distal epipharyngeal sensilla (dst) present, but not enclosed by furrow (sometimes dst absent)	Middle 4 setae of labro-epipharyengal margin consisting of 2 spatulate, broad inner (Mp) and 2 stout, narrow (p2) outer setae
***Anthrenus* Geoffroy, 1762**	Both hastisetae and spicisetae present	Head of hastisetae variable: 3 to more than 5 times as long as wide at the widest point	Present on nota II-III, incomplete on abdominal tergites I–IV; sometimes slightly visible on tergum V	Distal epipharyngeal sensillae (dst) arranged in one group of 6, but not enclosed by furrow (usually sensillae are in a faintly defined fusiform area)	Middle 4 setae of labro-epipharyengal margin consisting of 2 spatulate, broad inner (Mp) and 2 stout, narrow (p2) outer setae
***Ctesias* Stephens, 1830**	Both hastisetae and spicisetae present	Head of hastisetae more than 3 times as long as wide at the widest point	Absent on notum I, but distinct on nota II–III and abdominal terga I–VII (Figs 18 and 19); abdominal segment VIII without the suture or only remains of it can be observed	Distal epipharyngeal sensillae (dst) arranged in one group of 6 (in two longitudinal series of 3 sensillae), not enclosed by distinct furrow	Middle 4 setae of labro-epipharyengal margin consisting of 2 spatulate, broad inner (Mp) and 2 stout, narrow (p2) outer setae
***Globicornis* Latreille, 1829**	Both hastisetae and spicisetae present	Head of hastisetae less than 3 (e.g. *G. corticalis* and *G. emarginata*), or more than 3 times as long as wide at the widest point (e.g. *G. nigripes*)	Absent on notum I, but distinct on nota II and III as well as abdominal terga I–VIII (on segment VIII its form reminds thread-like carina)	Distal epipharyngeal sensilla arranged in two groups: one of two sensillae, and second of four sensillae; both groups completely enclosed/encircled by a furrow (except *G. nigripes* in which six distal sensillae are enclosed in one ring); besides in *G. corticalis* and *G. emarginata* apices of the epipharyengal rods are joined by a sclerotised transverse bar	Middle 4 setae of labro-epipharyengal margin consisting of 2 spatulate, broad inner (Mp) and 2 stout, narrow (p2) outer setae
***Megatoma* Herbst, 1792**	Both hastisetae and spicisetae present	Head of hastisetae more than 3 times as long as wide at the widest point	Smooth and distinct, present on nota I–III and abdominal terga I–VIII (absent on segment VIII in subgenus Pseudohadrotoma)	Distal epipharyngeal sensillae arranged in one group of 6 in two rows	Middle 4 setae of labro-epipharyengal margin consisting of 4 spatulate, broad setae both in inner (Mp) and (p2) outer setae
***Reesa* Beal, 1967**	Both hastisetae and spicisetae present	Head of hastisetae more than 3 times as long as wide at the widest point	Absent on notum I, but distinct and denticulate on nota II–III and abdominal terga I–IX	Distal epipharyngeal sensillae arranged in one group (enclosed in distinct ring) of 6	Middle 4 setae of labro-epipharyengal margin consisting of 2 spatulate, broad inner (Mp) and 2 stout, narrow (p2) outer setae
***Trogoderma* Dejean, 1821**	Both hastisetae and spicisetae present	Head of hastisetae less than 3 times as long as wide at the widest point	Not always well defined; if present usually absent on abdominal segment VIII, but in those rare instances when it is present, it is weak and interrupted at several points	Distal epipharyngeal sensillae arranged in one group (enclosed in distinct ring) of 4-6(7) or sometimes in two rings of 2 and 4	Middle 4 setae of labro-epipharyengal margin consisting of 2 spatulate, broad inner (Mp) and 2 stout, narrow (p2) outer setae

^3^ But only in late instar larvae (Zhantiev 2000) ^4^ According to Peacock (1993, p. 125, fig. 209)

**Table 2.3:** Continuation of the comparison of larval characteristics for some of the genera of Dermestidae co-occurring with *Ctesias serra*.

Character	# of stemmata	Antenna	Maxillary palp	Pretarsal setae
Genus				
***Dermestes* Linnaeus, 1758**	6^5^	Antennal segment 2 more than twice (3-4x) as long as segment 3; sensorium arising from apex of segment 2 and never terminates at apex of segment 3	4 segments	Equal
***Thylodrias* Motschulsky, 1839**	3	Antennal segment 2 much narrower and shorter than segment 1 and half as long as segment 3; sensorium arising from apex of segment 2 and terminates at middle of segment 3	4 segments	Unequal (anterior seta shorter than posterior one)
***Trinodes* Dejean, 1821**	6	Antennal segment 2 nearly as long and broad, as segment 1 and less than half as long as segment 3; sensorium arising from basal third of segment 2 and terminates almost at apex of segment 3	4 segments	Unequal (anterior seta shorter than posterior one)
***Attagenus* Latreille, 1802**	4–5^6^	Antennal segment 2 more than twice (3-4x) as long as segment 3; sensorium arising from apex of segment 2 and never terminates at apex of segment 3	4 segments	Probably variable – depends on species
***Anthrenus* Geoffroy, 1762**	6	Antennal segment 2 more than twice as long as segment 3; sensorium arising from apex of segment 2 and never terminates at apex of segment 3	3 segments	Variable – depends on species
***Ctesias* Stephens, 1830**	6	Antennal segment 2 more than twice as long as segment 3; sensorium in ventral position – only sometimes slightly extending above apex of segment 2	3 segments	Subequal
***Globicornis* Latreille, 1829**	6	Antennal segment 2 twice as long as segment 3; sensorium in ventral position, below the apex of segemnt 2	3 segments	Equal (e.g. *G. corticalis*, *G. emarginata* and *G. nigripes*^7^)
***Megatoma* Herbst, 1792**	5(?)^8^	Antennal segment 2 twice as long as segment 3; sensorium in ventral position, below the apex of segemnt 2	3 segments	Equal
***Reesa* Beal, 1967**	4	Antennal segment 2 not more than half as long as segment 3; sensorium in ventral position, below the apex of segemnt 2; antennal segment 2 with at least one seta (sometimes two setae)	3 segments	Subequal
***Trogoderma* Dejean, 1821**	5	Antennal segment 2 not more than half as long as segment 3; antennal segment 2 either without setae or with one-two seta(e)	3 segments	Variable – depends on species

^5^ According to Zhantiev (2000) *Dermestes depressus* lacks stemmata. ^6^ According to Beal (1991) ^7^ After Peacock (1993: p. 37) ^8^ According to Kadej (2017)

Moreover, because *Ctesias serra* inhabits quite similar habitats (= in or near spider webs, under loose bark, in old decayed wood) as some of the representatives of the genera *Anthrenus* (e.g. *A. fuscus* Olivier, 1789 or *A. museorum* (Linnaeus, 1761)), *Globicornis* (e.g. *G. corticalis* (Eichhoff, 1863) or *G. emarginata* (Gyllenhal, 1808)), *Megatoma* (e.g. *M. undata* (Linnaeus, 1758)) or *Trinodes* (e.g. *T. hirtus* (Fabricius, 1781)), I have identified morphological characters that could aid precise determination. The first characteristic that is useful in distinguishing *Ctesias serra* from other species is body shape. In *C. serra* it is constricted at abdominal terga I–III, and the body is broadest at notum III (ratio length to width 1.0:3.0), while in the rest of the genera there is no constriction. The body is broadest at abdominal segments IV–VI in *Anthrenus*, at notum III or abdominal segment I in *Globicornis* and *Megatoma* (ratio length to width 1.0:4.5), and at abdominal segment I in *Trinodes* (ratio length to width 1.0:2.5).

Also the integument colour is distinctly different in *Globicornis* (dark brown to black) and *Trinodes* (greyish with a sclerotised brown strip along the anterior and posterior margin of the tergite, enclosing a transverse membranous area on each side), while in *Ctesias* it is yellowish brown to brown (and thoracic terga (= nota I–III) sometimes with darker spots or patches). *Megatoma* is also yellowish brown (in some species have thoracic terga I–III with distinctly dark brown patches at sides, sometimes extending to middle on terga II and III). Only in *Ctesias* do the sterna I–VIII have a central median line with a strongly sclerotised and shiny strip.

Other differences involve morphology and the location of abdominal tufts of hastisetae. In *Ctesias* and *Anthrenus* they are situated behind abdominal terga and always on membranous areas, while in *Globicornis* and *Megatoma* they are located on sclerotised areas on abdominal terga (and never on membranes behind the terga). *Trinodes* do not have hastisetae. In *Ctesias* there are four tufts of hastisetae (on the membranous area behind the abdominal terga IV–VII), while in *Anthrenus* there are only three (on the membranous area behind the abdominal terga V–VII). Additionally, in *Trogoderma*-like Megatomini such as *Megatoma*, dense brushes of hastisetae are dark brown and compact (hastisetae are shorter and densely packed under the terga), while in *Ctesias* the hastisetae are lighter (= brown or yellowish brown), longer, and are loosely packed. For other features see Table 2. A key for identification of the genera of Dermestidae co-occurring with *Ctesias* is presented below.

A separate comment is required regarding pupae since, as for the larvae, there are no detailed descriptions of the morphology of this stage for *Ctesias* except for a brief description at the generic level by Donisthorpe (1897, p. 162, cf. Table 1). This genus now contains 26 species (Háva 2018). Here, for the first time, the morphological characteristics of the pupae of *C. serra* is presented. Lack of additional data for other species does not allow for any comparison with other representatives of that genus. Nevertheless, it is worthy to compare some pupal characteristics with available data of other pupa known within the Dermestidae.

Pupae of *C. serra* lack gin-traps. These occur in Dermestini Latreille, 1804 or Attagenini Laporte de Castelnau, 1840 and are thought to protect soft-bodied pupae from predators or parasites, like mites (Zhantiev 1976, Kiselyova and McHugh 2006). For this reason *C. serra* is similar to some pupae of Trinodini Casey, 1900, Thylodriini Semenov-Tian-Shanskiy, 1909 and Anthrenini Gistel, 1848, in which the gin-traps are either poorly developed or lacking. The pupae of the abovementioned taxa retain their larval exuviae to which they remain firmly attached via anchor setae. The same behavior was observed by Donisthorpe (1897, 1920) who reported that pupae of *C. serra* remain within the last exuvium which is interrupted from head to the last abdominal terga. The part of the dorsum that remains exposed is usually covered in long, soft or stiff hairs. The same can be seen on the dorsal side of the pupae of *Megatoma undata* (Linnaeus, 1758) shown by Kadej (2017, p. 65). Long hairs have not been observed on the dorsum of *C. serra* but these could have been lost before exanination of material.

It is noteworthy that the pupae of *C. serra* have two urogomphi-like processes on the IX abdominal segment. Typical urogomphi are mainly known from the larval stages of *Dermestes* Linnaeus, 1758, *Orphilus* Erichson, 1846 and *Thorictodes* Reitter, 1875 (Beal 1991). These characters are also present in pupae of *Dermestes*. However, it is also known that there are some genera that do not possess urogomphi as larvae, but do so as pupae (Kiselyova and McHugh 2006). Such situation has been observed in the larval and pupal stages of *Attagenus* Latreille, 1802 and *Novelsis* Casey, 1900. The presence of urogomphi in the pupa is probably an ancestral character. According to Kiselyova and McHugh (2006) their presence in *Dermestes* and in some Attagenini Laporte de Castelnau, 1840 may be a retained plesiomorphic trait. It is difficult to distinguish whether these two processes I have reported in pupa of *C. serra* are typical urogomphi and they may only a deformation of the pupa. To exclude atypical morphology or to confirm the presence of urogomphi, longer series of individuals are required to answer this question. If these processes are indeed typical urogomphi, than this observation in *Ctesias* contradicts the statement of Kiselyova and McHugh (2006) that the rest of Dermestidae lack urogomphi and pupate within the last larval exuvium. Regardless, the example of *C. serra* sheds new light on our knowledge of pupal stages and indicates the need for further studies of these unexplored stages. Kiselyova and McHugh’s analysis demonstrates how useful pupal characteristics can be in the investigation of phylogenetic relationships between genera. The need for further research is also justified by the example of the recently described pupae of *M. undata* (Kadej 2017). The pupae here do not have either urogomphi or processes, although as in *C. serra*, this species is classified within the Megatomini Leach, 1815. Therefore, it is also likely that pupal characteristics would be an excellent taxonomic tool for the Dermestidae. There is a great need to discover characteristics which may be useful taxonomic features for distinguishing genera or even enabling species identification. Thus, more taxonomic studies are needed in order to assess the value of different morphological characters of pupae in the Dermestidae.

### Key to identification genera co-occuring with *Ctesias* Stephens, 1830

**Table:** 

1	Urogomphi on IX abdominal tergite dorsally	***Dermestes* L., 1758**
–	Urogomphi on IX abdominal tergite dorsally absent	**2**
2	Abdominal tufts of hastisetae absent	**3**
	Abdominal tufts of hastisetae present	**5**
3	Body cylindrical, broadest at notum I; with caudal brush of long, slender setae	***Attagenus* Latreille, 1802**
–	Body compact, broadest at abdominal segment I, without caudal brush of long, slender set	**4**
4	Body uniformly light golden brown; posterior margin of terga with club-shaped setae; antennal segment 2 much narrower and shorter than segment 1 and half as long as segment 3; sensorium arising from apex of segment 2 and terminates at middle of segment 3	***Thyodrias* Motschulsky, 1839**
–	Body brown or greyish with sclerotised brown strip along anteriorand posterior margin of tergite, enclosing a transverse membranous area on each side; posterior margin of terga without club-shaped setae – black, long, stout spicisetae present; antennal segment 2 nearly as long and broad, as segment 1 and less than half as long as segment 3; sensorium arising from basal third of segment 2 and terminates almost at apex of segment 3	***Trinodes* Dejean, 1821**
5	Abdominal tufts of hastisetae on membranous area behind terga	**6**
–	Abdominal tufts of hastisetae on terga (never on membranous area behind terga)	**7**
6	Body broadest at notum III and constricted behind abdominal terga I–III; caudal brush of long, slender setae present; abdominal tufts of hastisetae on membranous area behind terga IV–VII; sterna I–VIII with central median line with strongly sclerotised and shiny strip	***Ctesias* Stephens, 1830**
–	Body broadest at abdominal terga IV–VI and not constricted; slender setae present on terga IX, but not so long as it is in *Ctesias*; abdominal tufts of hastisetae on membranous area behind terga V–VII	***Anthrenus* Geoffroy, 1762**
7	Antennal segment 2 twice as long as segment 3	**8**
–	Antennal segment 2 not more than half as long as segment 3	**9**
8	Terga brown to dark brown; abdominal tufts of hastisetae on terga V–VIII	***Globicornis* Latreille, 1829**
–	Terga yellowish brown (in some species thoracic terga I–III with distinctly dark brown patches at sides, sometimes extending to middle on terga II and III); abdominal tufts of hastisetae on terga VI–VIII	***Megatoma* Herbst, 1792**
9	Abdominal tufts of hastisetae on terga I–VIII; antecostal suture absent on notum I, but distinct and denticulate on nota II–III and abdominal terga I–IX; head of hastisetae more than 3 times as long as wide at the widest point	***Reesa* Beal, 1967**
–	Abdominal tufts of hastisetae on terga V (VI)–VIII; antecostal suture not always well defined; if present usually absent on abdominal segment VIII, but in those rare instances when it is present, it is weak and interrupted at several points head of hastisetae less than 3 times as long as wide at the widest point	***Trogoderma* Dejean, 1821**

## Supplementary Material

XML Treatment for Ctesias (s. str.) serra
